# Natural products as a means of overcoming cisplatin chemoresistance in bladder cancer

**DOI:** 10.20517/cdr.2020.69

**Published:** 2021-03-19

**Authors:** Ganeshkumar Rajendran, John A. Taylor, Benjamin L. Woolbright

**Affiliations:** Department of Urology, University of Kansas Medical Center, Kansas City, KS 66160, USA.

**Keywords:** Cisplatin, bladder cancer, natural products, apoptosis

## Abstract

Cisplatin remains an integral part of the treatment for muscle invasive bladder cancer. A large number of patients do not respond to cisplatin-based chemotherapy and efficacious salvage regimens are limited. Immunotherapy has offered a second line of treatment; however, only approximately 20% of patients respond, and molecular subtyping of tumors indicates there may be significant overlap in those patients that respond to cisplatin and those patients that respond to immunotherapy. As such, restoring sensitivity to cisplatin remains a major hurdle to improving patient care. One potential source of compounds for enhancing cisplatin is naturally derived bioactive products such as phytochemicals, flavonoids and others. These compounds can activate a diverse array of different pathways, many of which can directly promote or inhibit cisplatin sensitivity. The purpose of this review is to understand current drug development in the area of natural products and to assess how these compounds may enhance cisplatin treatment in bladder cancer patients.

## Introduction

Bladder cancer (BCa) is a common solid tumor with high rates of morbidity and mortality, especially in patients with advanced disease. Tumor stage is predicated on invasion into the bladder musculature and is linked to both treatments and patient outcomes^[1,2]^. Non-muscle invasive disease is treated with resection of the tumor and is typically followed by intravesical treatment with Bacillus-Calmette-Guerin (BCG) immunotherapy^[1]^. In contrast, muscle invasive disease is typically treated with radical cystectomy^[2]^. In addition, either adjuvant (after surgery) or neoadjuvant (before surgery) cisplatin-based chemotherapy is typically given to patients to reduce tumor burden and to treat potential metastatic disease^[1,3-6]^. A complete response to chemotherapy is associated with improved outcomes^[3,7]^. Unfortunately, up to 50% of patients are resistant to cisplatin-based chemotherapy or develop resistance over time^[8]^. Resistance is further problematic as cisplatin has a number of known toxicities, including cardiotoxicity, ototoxicity, and particularly nephrotoxicity that preclude usage^[9,10]^. This is extremely problematic in a patient population composed largely of elderly patients with high incidences of smoking as well as other co-morbidities. Treating patients that are unlikely to receive a benefit is thus inadvisable and simply increasing dosages in patients that are not initial responders is not safe due to toxicity. As such, advancing cisplatin-based chemotherapy remains a major avenue for improving patient outcomes in patients with muscle-invasive disease.

Natural products derived from plant and animal sources have long been a source of biologically active compounds. Natural product derived compounds such as the taxols, rapamycin, digoxin and more have successfully been translated to the clinic for various diseases; whereas a multitude of other compounds, or extracts from plant sources, remain under investigation for their noted biological activity. Many of these compounds have been reported to enhance the efficacy of cisplatin or block the toxicity in other organs^[11,12]^. Developing these compounds as a means for improving cisplatin therapy offers potential to offer cisplatin to a wider range of patients and improve patient response.

The purpose of this review is to understand the mechanisms that dictate the ability of cisplatin to block proliferation and enhance apoptosis in cancer cells and the mechanisms that result in cisplatin resistance. We will further discuss how compounds derived from natural products can potentially abrogate cisplatin resistance and improve therapeutic response.

## Cisplatin based chemotherapy in bladder cancer

Current chemotherapy regimens used for BCa include either MVAC (methotrexate, vinblastine, adriamycin, cisplatin) or GC (gemcitabine/cisplatin)^[3,4,7]^. Both are largely dependent upon response to cisplatin more so than the other compounds. Understanding the molecular mechanisms that determine cisplatin efficacy and predicting which patients will respond to cisplatin treatment remains a major topic of interest in BCa treatment.

### Molecular mechanisms

**Figure 1:**
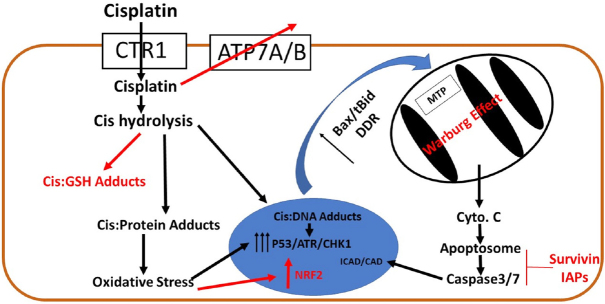
Mechanisms of cisplatin toxicity and resistance. Cisplatin is transported either actively by copper transporters such as CTR1 across the plasma membrane or passively through diffusion. Cisplatin can be actively transported out of the cell by metal transporters such as ATP7A and ATP7B and thus overexpression of these transporters is a mechanism of resistance. Cisplatin is hydrolyzed to its active form in the cytosol where it can bind proteins inducing oxidative stress or can be detoxified via cellular antioxidants like GSH. Active cisplatin binds purines on DNA which results in activation of p53 and the DNA damage response. P53 target genes like Bax and cellular stress from oxidative damage activate MOMP formation in the mitochondria, resulting in release of cytochrome c and activation of the apoptosome. Caspase3/7 cleave ICAD which release CAD and cleaves DNA leading to apoptosis. Black arrows notate events that promote toxicity, red arrows notate events that promote resistance. CTR1: copper transporter 1; Cis: cisplatin; GSH: glutathione; Bax: Bcl2 associated X-protein; tBid: truncated - BH3 domain interacting-domain death agonist; ATR: ataxia telengeicstasia and Rad3 related protein; CHK1: checkpoint kinase 1; Cyto C: cytochrome C; MTP: mitochondrial permeability transition pore; IAPs: inhibitors of apoptosis proteins; NRF2: nuclear factor erythroid-derived 2-like

We will focus on the primary understood mechanisms of cisplatin efficacy and resistance, and how they relate to cancer treatment in the bladder^[13,14]^. Cisplatin uptake is actively mediated by the copper receptor CTR1^[15,16]^. Cellular uptake is required for cisplatin efficacy, as it is largely inactive until it reaches the cytosol where it is biologically activated via aquation^[17,18]^. The active form of cisplatin can adduct or otherwise interact and bind with proteins, DNA, and cellular antioxidants including glutathione (GSH) or free cysteines on proteins such as metalliothionein. Cisplatin adducts are the major source of cellular damage caused by cisplatin, especially cisplatin-DNA adducts formed on purines, particularly guanine^[18,19]^. The formation of DNA adducts also leads to multiple DNA damage response pathways resulting in the activation of ataxia telangiectasia and Rad3-related protein (ATR)/ATM serine/threonine kinase (ATM) and checkpoint kinase 1 (Chk1) and checkpoint kinase 2 (Chk2)^[20]^. ATR/ATM/Chk1/Chk2 activation initiates pro-apoptotic signaling pathways mediated by p53 that converge on the mitochondria, although p53 independent cell death has also been noted, consistent with the fact that cisplatin is used in both p53 WT and p53 mutant tumors^[21,22]^. Cisplatin is often more effective in p53 mutant cells further highlighting the dependency of cisplatin on DNA damage to induce cytotoxicity^[9,21]^. P53 is known to mediate the effects of chemotherapeutics differently between tissues and thus fully understanding how p53, commonly mutated in BCa, interacts with cisplatin in BCa, remains critical^[23]^. Finally, to execute cell death, mitochondrial stress results in release of components critical to the apoptosome including cytochrome c and apoptosis inducing factor (AIF) and this results in the activation of executor caspases (3/7) and apoptotic cell death through irreversible DNA cleavage^[14]^ [Figure 1]. Interplay between Bcl-2 family protein members such as Bid, Bax, and Bcl-XL as well as inhibitor of apoptosis (IAP) family members such as survivin further modulate the apoptotic response in cancer cells and are widely cited targets of natural products associated with chemotherapy.

While apoptosis is the most widely cited cause of cell death after cisplatin treatment, it should be noted that clinical specimens treated with cisplatin do not necessarily show pure apoptosis^[24]^. Furthermore, while *in vitro* experiments lead to activation of caspases, apoptosis may not be the predominant form of cell death at many doses of cisplatin *in vitro*^[24,25]^. Quantitative assessments of both necrosis and apoptosis found that in breast, gastric, and prostate cancers, levels of apoptosis induced by cisplatin were substantially less than levels of necrosis^[24]^. This may be due to inherent resistance to apoptosis in many cancer cells, and thus cell death input is eventually converted to cellular necrosis instead of cellular apoptosis as has been observed in other models^[26]^. As cellular necrosis is known to stimulate the immune environment via release of pro-inflammatory intracellular mediators, cisplatin-induced necrosis may benefit immunotherapy in the future by amplifying and sustaining an immune response^[27,28]^. Active trials combining immunotherapy and chemotherapy in BCa have thus far proved disappointing, but more trials are required to optimize this interaction^[29]^. It may be possible to stimulate an inflammatory environment while also killing tumor cells by initiating treatment with cisplatin and following up with sustained immunotherapy.

### Mediators of cellular resistance to cisplatin

Multiple mechanisms of resistance to cisplatin have been well established. These mechanisms follow the typical pattern of resistance mechanisms associated with alkylating agents such as cisplatin. This includes active transport into the cell, detoxification of cisplatin, altered DNA damage responses that prevent pro-apoptotic cellular signaling, direct inhibition of apoptosis, active export out of the cell, and inhibition of cellular energetics associated with Warburg metabolism (aerobic glycolysis). We will discuss these individual mechanisms and the mechanisms by which they reduce cisplatin efficacy.

#### Active transport of cisplatin

Cisplatin contains a central platinum that mediates a number of the biochemical properties of the compound. Transporters that control metal import and export from the cell, particularly copper transporters, also transport cisplatin and can result in resistance due to alterations in import and export of the drug^[16]^. These transporters are differentially regulated in many cancers where cisplatin is used clinically, and their regulation is associated with both resistance to therapy and off-target toxicity^[30-32]^. Moreover, laboratory models of ATP7A or ATP7B metal transporter overexpression result in resistance, consistent with the idea that active transport *out* of the cells can be just as important a mechanism as active transport *into* the cell^[32]^. These data coalesce around the idea that cisplatin transport is far from passive, and that uptake and export must be factored into both patient response and potential adverse events.

#### Detoxification of cisplatin intracellularly

Cisplatin resistance or susceptibility likely occurs to some degree in all cells, tumor or benign, at the level of detoxification. Increased levels of glutathione (GSH), activation of the transcription factor nuclear factor erythroid 2-related factor 2 (Nrf2), or indirect activation of Nrf2 via silencing of the Nrf2 binding partner Kelch Like ECH Associated Protein 1 (Keap1), upregulation of metal binding proteins such as metallothionein, and increased levels of thiol compounds can result in direct protection against cisplatin via detoxification^[33-37]^. All of these mechanisms are thought to work in part through direct detoxification of active cisplatin. GSH acts as a cellular sink for cisplatin and is easily replaced through synthesis of new GSH yielding continuous detoxification of cisplatin^[33,35,36,38,39]^. Depletion of GSH prior to cisplatin treatment with agents like buthionine sulfoximine dramatically enhances cisplatin efficacy; although as this would occur throughout the body, this mechanism is not a rational means for improving cisplatin clinically as it would also enhance cisplatin toxicity in susceptible tissues that were also depleted of GSH^[38,39]^. Metal binding proteins such as metallothionein are thought to directly bind the platinum in cisplatin via interactions with the many thiol groups present on cysteine rich metallothioneins, leading to direct detoxification. Blocking metallothionein expression would globally increase toxicity in all cells though, and thus is not a viable means for enhancing cisplatin. Notably, the ROS responsive transcription factor nuclear factor erythroid 2-related factor 2 (Nrf2) can enhance synthesis of GSH through upregulation of GSH synthesizing proteins such as GCLC and GCLM^[40]^. Similarly, Nrf2 directly upregulates metal-binding proteins and phase I and phase II transporters that detoxify or otherwise prevent cisplatin toxicity^[40]^. This limits the amount of DNA/protein damage directly and leads to resistance. Many cancers are considered Nrf2 “addicted” and rely on Nrf2 activation^[41]^. A number of naturally derived compounds are putative Nrf2 activators and thus Nrf2 activation remains an understudied but likely mechanism through which many compounds can either block or promote cisplatin toxicity depending on their actions on Nrf2.

#### Mutations in DNA damage repair

A number of mutations and/or alterations in expression of genes associated with nucleotide excision repair (NER) can alter response to cisplatin. In BCa, recent research has focused on *ERCC2* mutations as a major mediator of cisplatin sensitivity^[42]^. *ERCC2* is normally responsible for NER of bulky adducts such as what is observed with cisplatin^[43]^. Mutation of *ERCC2* confers sensitivity to cisplatin *in vitro* in cell lines and *ERCC2* mutations are enriched significantly in responders to cisplatin^[42,44]^. Importantly, ablation of NER activity occurs with the majority of *ERCC2* mutations, and experimentally initiated mutations had highly similar activities, consistent with the idea that most mutations of *ERCC2* would convey susceptibility to cisplatin^[42,44]^. These data concur with the major mechanism of cisplatin-mediated tissue injury being the formation of DNA adducts and the subsequent cellular response, as loss of NER would enhance cisplatin-DNA adduct formation due to lack of repair, resulting in more potent cell death signaling. Monitoring *ERCC2* status remains a major potential biomarker of response to cisplatin and is a likely area of future clinical trials prospectively evaluating response to cisplatin based on *ERCC2* status.

In contrast to *ERCC2*, *ERCC1* positivity has highly variable results between studies. An initial study suggested low ERCC1 expression is associated with prolonged survival; however, a separate study two years in a larger cohort indicates patients that have no expression of ERCC1 have prolonged survival versus though patients that have some expression of ERCC1^[45-47]^. It is possible that some degree of ERCC1 expression is required. Notably, it has been suggested this may not be related to cisplatin efficacy; although, other papers indicate that ERCC1 negative tumors benefit from neoadjuvant gemcitabine plus cisplatin-based chemotherapy^[46-50]^. Given the major discrepancies with ERCC1, it is unclear what data are correct; however, it may still be useful to distinguish what delineates the difference in these studies and determine more completely the role of ERCC1.

Mutations in base excision repair (BER) may also contribute to cisplatin effect. The BER protein Apurinic/apyrimidinic endonuclease 1/redox factor-1 (APE1/Ref-1) has recently been identified as a target for enhancing the efficacy of cisplatin in bladder cancer^[51]^. APE-1 is upregulated in BCa which results in activation of transcription factors such as NF-κB that upregulates pro-survival proteins^[52]^. Inhibition of APE-1 with the novel compound E3330 increases the efficacy of cisplatin in addition to killing cells on its own^[53]^. Notably though, it remains unclear if this is specifically related to BCa, or the ability of E3330 to block BER, especially as APE1 may also regulate Nrf2^[54]^. Studies in breast cancer indicate E3330 enhances cisplatin resistance via blocking the pro-apoptotic response associated with DNA repair, and in contrast, suggests the lyase domain of polymerase ß is irrelevant, whereas knockdown of XRCC1 results in sensitivity to cisplatin due to increased DNA damage^[55]^. Whether or not these mechanisms are universal between cancers thus remains in doubt; however, in BCa, it appears that BER may be a target for improving cisplatin-based therapy. It needs to be determined if the actions are of APE-1 and other BER proteins are directly through base excision repair, or instead through regulation of transcription factors such as Nrf2 that can also regulate the effects of cisplatin.

#### Modulators of the Warburg effect

Otto Warburg originally coined the idea that cancer cells primarily use glycolysis to produce ATP for energy^[56]^. Modern interpretation has emphatically demonstrated cancer cells have highly functional mitochondria and predominantly use oxidative respiration, and that while cancer cells upregulate many of the enzymes associated with glycolysis, the increased level of glucose uptake and glucose metabolism generate not only ATP but also oxidative biomass and metabolic intermediates which are equally if not more important^[57,58]^. A number of compounds that act on the Warburg Effect through inhibition of critical Warburg enzymes such as glucose transporter 1 (GLUT1), pyruvate dehydrogenase kinases (PDKs) or pyruvate kinase M2 (PKM2) also enhance cisplatin-based therapy, including in BCa^[58-60]^. Mechanisms delineated in other cancers indicate this is likely due to increased production of ROS by damaged mitochondria that are forced to use mitochondrial metabolism to generate the necessary biological intermediates^[57,58,61]^. Natural products targeting these enzymes have been discovered, and may serve as a source of chemotypes that can be further improved for targeting the Warburg Effect and overcoming cisplatin chemoresistance^[57,62]^.

#### Direct resistance to cisplatin induced apoptosis

The primary mechanism noted in studies of cisplatin induced cell death is mitochondrially mediated caspase induced apoptosis^[13,14]^ [Figure 1]. Because apoptosis is a tightly controlled process mediated by both positive and negative regulators, cellular changes in the apoptotic process are not uncommon and can promote resistance to apoptosis^[7,43]^. Bcl-2 family proteins such as survivin and Bcl-xL promote resistance and these proteins are commonly upregulated in cancer^[63]^. In contrast, pro-apoptotic proteins such as Bax and Bid that initiate the mitochondrial permeability transition pore (MTP) necessary for release of pro-apoptotic factors from the mitochondria are commonly downregulated or dysregulated in cancer such that there is implicit resistance to apoptosis^[14,63]^. This may be a potential reason for the observation that, in spite of the focus on apoptosis as a major mediator of cisplatin toxicity, clinical cisplatin administration is consistent with mixed cell death (apoptosis and necrosis)^[24]^. A number of pathways that amplify the apoptotic signal, such as the c-Jun N-terminal kinase (JNK)-mediated pathway have been implicated in cisplatin toxicity as well and likely amplify upstream effects^[64]^. These pathways are confined to normal cells as JNK also amplifies cell death signals in tubular kidney cells exposed to cisplatin^[65]^. Natural products have routinely been shown to either amplify or inhibit associated pathways and thus may be useful for either treatment of cisplatin induced cytotoxicity or amplification of cisplatin induced anti-cancer activity.

#### Cisplatin based chemotherapy in bladder cancer - predicting response in molecular subtypes

While cisplatin remains standard of care only 30%-50% of patients respond and many have adverse events during the course of treatment^[66,67]^. Identification of biomarkers that can rapidly and effectively identify those patients that will respond is imperative. With the advent of molecular subtyping, a number of studies have demonstrated that BCa segregates into distinct groups based on genomic profiling^[68-75]^. Multiple groups have now established there is considerable difference between how these groups respond to cisplatin based, or other chemotherapy^[71,76]^. Tumors defined as luminal or basal subtype respond well to cisplatin; whereas recent information suggests neuroendocrine type or genomically neuroendocrine-like tumors and claudin-low tumors are poor responders to cisplatin with higher mortality rates^[71,72,74,76,77]^. Patients that respond well to cisplatin tend to have mutations in *ERCC2* as mentioned above and high tumor mutational burdens^[42,44,47]^. Ultimately, these biomarkers are largely reflective of mechanisms that have been demonstrated in tumor cells and in human patients. Novel biomarkers that can improve which patients are designated for cisplatin-based chemotherapy are sorely needed. This would allow precision medicine initiatives aimed at reducing toxicity and improving therapy with all compounds, including natural products.

While this is not an exhaustive list of the mechanisms of cisplatin resistance, these mechanisms cover the majority of the known mediators. Cancer stemness and other similar factors are also highly associated with chemoresistance but, typically, stemness and other such forms of resistance directly involve those mechanisms listed above in some capacity. Understanding how current compounds affect these pathways as well as how to affect these pathways with novel compounds or synthetic derivatives remains critical to treating patients effectively with chemotherapy.

## Natural products - a validated source of biologically active compounds

Natural products have previously produced agents that have been used in multiple ways to treat cancer, including as direct chemotherapeutics, chemo-sensitizing agents, and agents that prevent chemotoxicity^[78-82]^. Notably, mechanistic detail in this area has demonstrated that many of these compounds activate or repress some of the same pathways. At the same time, consensus mechanisms are commonly lacking. This is due to the fact that many of the compounds likely have multiple effects. As such, some compounds may promote apoptosis, but potentially through different mechanisms in different cell lines. Due to the sheer number of agents currently under investigation, we are going to focus on classes of drugs that could potentially synergize with cisplatin to overcome drug resistance and potential mechanisms, especially those with recently developed novel compounds. We have provided a Table [Table 1] to given a broad overview of the compound described in this section as well as their proposed methods of action and sources. This table should serve as an introductory guide to following compounds.

**Table 1:** Natural product derived inhibitors with potential synergy with cisplatin

Class	Compounds	Source	Mode of action
HSP90 inhibitors	17DMAG 17AAG Novobiocin	*Streptomyces* Species	Inhibit *AKT, B-Raf, etc…* via direct downregulation of proteins due to lack of chaperone mediated folding
Flavone & Isoflavones	Geninstein	Plant Derived	Induce apoptosis
	Tangeretin	Citrus Peels	Potentiate or synergize with cisplatin in BCa Genistein is a phytoestrogen and agonist of estrogen receptor
Napthoquinones	Shikonin Alkannin	*Lithospermum erythrorhizon*	Inhibition of *PKM2* reverses Warburg Effect and produces antiproliferative & pro-apoptotic changes associated with pyruvate metabolism
Anthrocyclines	Adriamycin Epirubicin	*Streptomyces* Species	Inhibits topoisomerase II resulting in catastrophic DNA damage
	Evodiamine	Plant Derived	Evodiamine is a dual inhibitor of topoisomerase I and II
Allyl disulfides	(di & tri) allylsulfides	Garlic Derived	Induce apoptosis, activates caspases, reduce phosphorylation of CHK proteins, prevents resolution of double -strand breaks through an *ATR/ATM* dependent mechanism
Curcumin and curcumin derivatives	Curcumin	Plant (Turmeric) Derived	Induces apoptosis Downregulating anti-apoptotic proteins such as Bcl2 & survivin. Upregulating Pro-apoptotic protein e.g., Bax. Prevent upregulation of *COX2* Reduction in PGE2
Vitamin C	Ascorbic Acid	Typically plant derived	ROS-induced cell death May also downregulate *HIF1A*

Summary table of natural products, their source and mechanism of action. Numerous natural products have potential use in BCa. COX-2: cyclooxygenase; PGE2: prostaglandin E2; CHK: checkpoint kinase; PKM2: pyruvate kinase M2; AKT: protein kinase B; B-Raf: serine/threonine-protein kinase B-Raf; ATM: ataxia telangiectasia mutated; ATR: serine-threonine protein kinase ATR; ROS: reactive oxygen species; HIF1A: hypoxia inducible factor 1A

### HSP90 inhibitors

Heat-shock protein 90 (HSP90) is a chaperone protein that stabilizes proteins necessary for tumor growth including protein kinase B (AKT), B-raf, and more^[83]^. HSP90A has routinely been demonstrated to be critical to cancer growth. Geldanamycin was the initial HSP90 inhibitor and was originally isolated from *Streptomyces* strains of bacteria and is thus a bacterially derived natural product. Natural product derived compounds such as geldanamycin and its analogues 17DMAG, 17AAG, and the more targeting HSB90 have proven successful in laboratory models but have limited potential clinically due to on-target toxicity associated with inhibition of all HSP90 isoforms^[83,84]^. Efforts aimed at specifically inhibiting HSP90 have yielded novel compounds that can inhibit BCa proliferation, but unfortunately led to the understanding that this induces a heat-shock response that is deleterious to the anti-cancer activity associated with HSP90 inhibition. In contrast, efforts to inhibit the C-terminus of HSP90, or efforts to inhibit the N-terminus of the ß form of HSP90 specifically have yielded inhibitors that may circumvent the heat shock response while also yielding efficacious therapeutics^[84-86]^. Novobiocin is a bacterially derived HSP90 inhibitor that targets the C-terminus of HSP90^[87]^. Novobiocin and derivatives do not initiate the heat shock response to the same degree and thus may be more viable means for inhibiting HSP90 long-term^[84,87]^. These compounds are derived from current HSP90 inhibitors and as such, the relative improvement seen with additional medicinal chemistry efforts may result in beneficial compounds. This is evidenced by prior studies indicating 17-DMAG can effectively combat cisplatin resistance in CD44^+^ BCa causing stem-like cells^[88]^. The exact mechanism remains undetermined though, as HSP90 inhibitors can affect multiple pathways simultaneously given their broad range of protein targets. Further studies examining whether these compounds can functionally improve cisplatin-based therapy are warranted in HSP90 inhibitors with reduced toxicity.

### Flavones and Isoflavones

A number of flavones and isoflavones have anti-cancer activity. Many of these have direct anti-apoptotic activity through activation of caspases^[89]^. A few notable examples are listed here. Genistein is a well-known plant-derived isoflavone and phytoestrogen that blocks BCa proliferation with multiple potentially relevant mechanisms^[90]^. Laboratory investigation of genistein indicates it is likely to induce apoptosis through actions on the intrinsic apoptotic pathway in addition to downregulation of nuclear factor nuclear factor kappa-light-chain-enhancer of activated B cells (NF-κB) in BCa^[90]^. Mechanistic study on genistein indicates it may actually be significantly more complicated as it also is a full agonist of estrogen receptor ß and has significant estrogenic activity, i.e., it is a phytoestrogen^[91]^. Prior research with regards to estrogen receptors suggest there may be anti-apoptotic activity tied to estrogen receptor blockers in BCa that further complicates the mechanism^[92]^. In spite of these data, some epidemiological evidence points towards estrogen as potentially protective against BCa, as women experience lower rates of BCa and some aspects of estrogen production are associated with reduced overall risk for BCa occurrence^[93,94]^. It may be that genistein, or genistein derived synthetic products, could be used to prevent cancer, especially in female patients that are post-menopausal. In order for this to happen, considerably more understanding of the role of estrogen and genistein in BCa would be needed.

Tangeretin is a flavone isolated from citrus peels with proven anti-cancer activity against BCa and also is synergizes with other established compounds such as adriamycin^[95]^. In spite of this, it is not well established how tangeretin induces apoptosis although some reports indicate mitochondrial dysfunction is critical to the apoptotic process similar to cisplatin^[96]^. Studies determining how tangeretin and other similar flavonoids with high abundance in citrus fruit interact with cisplatin and other critical chemotherapeutics are necessary to pinpoint dosing regimens and mechanisms. A number of other flavone or flavone derivatives have been put forth as potential chemotherapeutic agents; however, it should be noted that many of these compounds suffer from very poor pharmacology that ultimately prevents their usage in the clinic. As of yet there is no clinical evidence that flavones or isoflavones could potentiate or synergize with cisplatin in BCa, although, given its success in other areas, this may be a fruitful area for combinatorial drug research that deserves further investigation.

### Napthoquinones

Shikonin and its enantiomer alkannin are napthoquinones derived from *Lithospermum erythrorhizon* in addition to some *Alkanna* plant species with noted anti-cancer activity^[59]^. Shikonin has direct anti-apoptotic activity in BCa cells and in xenograft tumor models and synergizes with cisplatin^[59,62]^. The mechanism appears to be through inhibition of PKM2^[59]^. PKM2 inhibition reverses the Warburg Effect and thus produces anti-proliferative and pro-apoptotic changes by altering the production of both ATP and oxidative biomass associated with pyruvate metabolism^[97]^. Notably, this effect has been shown to be non-responsive to the classical apoptosis inhibitor z-VAD-fmk and thus some doses of cisplatin may produce necroptosis rather than apoptosis^[98]^. Similar effects were found with other synthetic compounds acting on Warburg associated pathways in BCa indicating blockade of Warburg metabolism may be an effective means for treating BCa patients^[58,60]^. Importantly PKM2 is known to be upregulated in patients with BCa that are resistant to cisplatin, indicating this may be a common mechanism of resistance in patients^[98]^. This is an active area of novel research and both shikonin and other synthetic derivatives may be able to overcome chemoresistance^[99,100]^.

Anthracyclines*:* Anthracyclines are a class of drugs that inhibit topoisomerase II which results in cancer cell death due to catastrophic DNA damage. Possibly most notably is adriamycin, a component of one of the major chemotherapeutic regimens currently in use for BCa (dose dense MVAC). Adriamycin is directly derived from daunorubicin, a compound found in large quantities in *Streptomyces* bacterial strains. Adriamycin is thought to directly induce apoptosis in BCa cells through inhibition of topoisomerase II, eventually leading to cellular apoptosis due to uncontrolled DNA strand breakages. Adriamcyin improves cisplatin presumably through amplification of the apoptotic signal and enhanced DNA stress associated with DNA damage. Other anthracycline compounds such as epirubicin and 2”R)-4’-O-tetrahydropyranyl-doxorubicin (THP) have extensively been investigated for the potential use as an instillation in superficial BCa tumors^[101,102]^. Attempts at using intravesical epirubicin demonstrated that it did not improve Bacillus-Calmette-Guerin monotherapy^[103]^. Similarly, epirubicin alone fails to outperform BCG monotherapy on its own, although some effect with epirubicin alone was noted and the drug was well tolerated^[104-106]^. Given the prior success with adriamycin and known toxicity associated with topoisomerase II inhibitors future efforts should be aimed at developing inhibitors that are potent but with reduced toxicity. A number of new topoisomerase I and II inhibitors are currently being developed, some of which are derivatives of natural products^[107]^. Evodiamine is a plant derived dual inhibitor of topoisomerase I and II, demonstrating efficacy against a number of tumor cell types including BCa; although notably, the proposed mechanism in BCa was attributed to inhibition of mTOR/S6K1 resulting in apoptosis^[108,109]^. *In vitro* IC50s for evodiamine are fairly high though, and thus improved compounds may also improve clinical merit. Molecular efforts aimed at understanding how to reduce toxicity, and synthetic efforts aimed at developing improved compounds with increased potency and reduced toxicity are necessary.

### Allyl disulfide and other garlic derivatives

Compounds derived from garlic have potent anti-cancer activity, particularly diallyl disulfide, and triallyl disulfide. Early studies using non-orthotopically implanted murine tumors indicate large doses of garlic derivatives block cancer growth with dose limiting toxicity^[110]^. This activity is likely attributable to diallyl and triallyl disulfide as they directly induce apoptosis in BCa though activation of caspases^[111,112]^. In addition to apoptosis induction; allyl disulfides also reduce expression of metallomatrix proteinases which slows proliferation and invasion of BCa EJ cells which may contribute to their anti-tumorigenic effect^[113]^. Perhaps more importantly, diallyl sulfide induces phosphorylation of CHK proteins and potentially prevents resolution of double strand breaks through an ATR/ATM dependent mechanism^[114,115]^. As cisplatin also works through the formation of double strand breaks; and *ERCC1* mutation is known to enhance cisplatin therapy, it is possible that synergy could be observed between diallyl/triallyl disulfide and cisplatin. Garlic derivatives and cisplatin were found to synergize in gastric cancer; although, this was postulated to function through attenuation of Nrf2, a mechanism which has not been investigated in BCa with regards to garlic or garlic derivatives^[116]^. Even still, diallyl disulfide and other garlic derivatives may be highly efficacious, especially in cancers that are already known to be sensitive to cisplatin in human patients if the mechanism is ERCC1 dependent. Given the wide range of currently stated mechanisms, it is unclear what the exact mechanisms of diallyl disulfide induced apoptosis is, but it is consistently noted to block cancer proliferation induce apoptosis. Furthermore, relevant concentrations take extremely high doses that may not be reachable clinically in patients before the onset of toxicity.

### Curcumin and Curcumin derivatives

Curcumin is a plant-based compound derived from turmeric and a potent inhibitor of BCa cancer growth. Curcumin induces apoptosis directly by downregulating anti-apoptotic proteins such as Bcl-2 and survivin, and upregulating pro-apoptotic proteins such as Bax^[117]^. Perhaps more importantly, curcumin inhibits the formation of superficial tumors in a rat model of orthotopic BCa carcinogenesis^[117]^. Similar to other compounds derived from natural products, mechanisms other than apoptosis have been suggested. Recent work in the BCa field indicates curcumin may block BCa progression through mechanisms not previously appreciated. Curcumin can prevent upregulation of cyclooxygenase-2 (COX-2) which results in reductions in prostaglandin E2 (PGE2) as COX-2 is the primary metabolizing gene for the production of PGE2^[118,119]^. PGE2 levels promote tumor formation in BCa and other drugs targeting PGE2 production have demonstrated efficacy in BCa, including potentiation or improvement of cisplatin-based therapy^[8,120-123]^. Importantly, whether this is directly due to inhibition of PGE2 production or due to other factors remains debatable, and thus, it is likely that if curcumin does inhibit PGE2 this is not the only mechanism through which it can block BCa proliferation. Blockade of PGE2 may be an important means through which curcumin could be used therapeutically in BCa as studies have demonstrated PGE2 is both involved in BCa tumor progression and cisplatin chemoresistance. Specifically, PGE2 levels contribute to a cancer stem cell like phenotype that is known to be resistant to cisplatin-based therapy though multiple mechanisms, as listed above^[8]^. Evidence indicates curcumin can directly prevent BCa proliferation in addition to the indirect evidence that curcumin-mediated blockade of PGE2 production would also have beneficial effects^[8,124,125]^. Curcumin improves both cisplatin based chemotherapy and gemcitabine based therapy as well^[126,127]^. Advancement of curcumin is unlikely as curcumin pharmacokinetics indicate it is rapidly metabolized when given orally and bioavailability is poor^[128,129]^. Novel curcumin derivatives with improved bioavailability are actively being developed, but must overcome major issues with bioavailability while retaining their anti-apoptotic effects if they are to be used successfully clinically. These studies may also be useful for determining the effects of PGE2 on BCa.

### Vitamin C

A number of essential vitamins derived from different natural products have been investigated for anti-cancer activity. Oral doses of ascorbic acid (Vitamin C) do not produce systemic levels of Vitamin C consistent with anti-cancer activity due to active uptake by SLC23A1/SLC23A2 and high levels of excretion of non-absorbed Vitamin C^[130,131]^. Vitamin C is known to kill cancer cells at levels obtained observed during intravenous delivery of vitamin C, however^[130-132]^. The mechanism is thought to be through generation of hydrogen peroxide via an ascorbate radical formed during Vitamin C metabolism^[133]^. This radical is only minimally formed in blood which spares many blood cells normally damaged by chemotherapeutics. Alternate mechanisms including downregulation of hypoxia inducible factor 1α have also been proposed; although, how these mechanisms would directly kill cancer cells remains undetermined, whereas, increased levels of ROS are known to induce cell death^[134]^. Pharmacological levels of vitamin C are toxic to BCa cell lines which may occur in part through methylation of DNA and increased levels of 5-hydroxymethylcytosine which prevents malignancy in addition to established effects on ROS production^[135]^. No study has directly tested synergy between cisplatin and Vitamin C as of yet; however, prior work indicates vitamin C can improve carboplatin-based therapy for ovarian cancer in laboratory models and was shown to prolong overall survival and time to disease recurrence in a small trial, although the prolonged survival did not meet statistical significance^[132]^. Clearly, given the solid safety profile of Vitamin C and its prior successes, it may be a viable means of improving cisplatin-based chemotherapy, or alternatively, improving therapy for patients that need less toxic chemotherapy regimens such as those that are ineligible for cisplatin.

It should be noted that many of the above chemicals are present in higher quantities in some specific diets designed to promote human health. One of the more common diets containing high quantities of plants and plant derivatives that includes isoflavones, garlic and garlic derivatives and other natural products is the Mediterranean Diet. This diet is associated with reduced risk of BCa in some analyses^[136,137]^. While oral consumption of these agents is likely to result in extensive metabolism and produce far lower systemic concentrations than direct administration, some benefit may be yielded from consistent intake of the diet. If this intake is related to natural product consumption and chemo-preventative effects, then understanding and isolating these specific agents or understanding their combined effects may be beneficial to treating BCa patients.

### Natural products and other forms of chemotherapy

While cisplatin remains the mainstay chemotherapy for muscle invasive disease, it should be noted that immunotherapy in the form of BCG and immunotherapy in the form of checkpoint inhibitors are critical treatments for BCa in the modern era. Checkpoint inhibitors function through removing the blockade on T cells and re-establishing immunity; however, dysregulated inflammation is a known promoter of BCa^[138,139]^. As checkpoint inhibition as a therapeutic remains in its infancy, little information is available in this regard. Notably though, garlic derivatives are known to promote BCG effects and may improve the effects of other immunotherapy agents as well^[140]^. Similarly, reductions in PGE2 by curcumin may reduce the immunosuppressive environment and improve checkpoint inhibition. Given the diverse set of mechanisms associated with natural products, it will be imperative to define any potential benefit these agents may provide with immunotherapy.

## Conclusion

Natural products have been a major source of chemotherapeutic drugs and will likely continue to be so. The number of therapeutics or chemotypes derived from natural products currently under study is substantial and far outweighs those discussed here. Both cisplatin and many of the discussed natural products have multiple mechanisms which may complicate our understanding of how to effectively dose these compounds and generate synergy with cisplatin. One thing missing from many of these agents are solid, high quality pharmacokinetic/pharmacodynamic studies that generate the data required for proper dosing of these compounds. These studies may further illuminate how we can alter structure-activity relationships of these compounds to improve their efficacy *in vivo* and duplicate the positive affects we have observed. The establishment of effective *in vivo* doses will then allow for more detailed mechanistic studies that target those concentrations that are achievable and effective *in vivo.* Increasing our understanding of how these compounds work is critical to developing synthetic derivatives that have improved pharmacokinetics and greater efficacy. Moreover, understanding the molecular basis of chemoresistance will allow us to position new drugs appropriately in those patients most likely to be responsive to chemotherapy. In summary, future studies aimed at refining current natural product derived compounds towards those that target specific aspects of cisplatin chemoresistance will be the most likely route to generate novel compounds that can improve patient chemotherapy.

## Declarations

### Copyright

© The Author(s) 2021.
